# Bourgery on the Anatomy of the Spleen

**Published:** 1842-10

**Authors:** 


*Bourgery on the Anatomy of the Spleen.*—M. Bourgery considers the spleen to consist of ten component parts:—1. Vesicular membranes; 2. blood-vessels; 3. floating vascular corpuscles; 4. a granulo-capillary ground; 5. a splenic liquid; 6. splenic glands; 7. lymphatic vessels; 8. nerves; 9. cellular tissue; 10. the enveloping membrane of the spleen.

The splenic vesicles are uniformly spread over the whole extent of the organ, are of an irregular polyhedral, and, when fully injected, of a spheroidal or oval shape, varying in size in different animals, and also in the same spleen. In man their mean diameter is from 1 to 1¼ millimetre. Each vesicle does not form a simple cavity, its walls being traversed by vessels which form projections in the interior, covered by the lining membrane. In consequence of this arrangement the cavity is divided into cells, at the bottom of which the minute glands and capillary vessels are seen in relief. There are orifices in vesicles of two sorts:—1. Orifices of communication more or less irregularly circular, whose edges are thin, and formed by a fold of the membrane which lines the cavity. Their diameter is from a quarter to half of that of the vesicle. There are one, two, or three orifices in each vesicle; and to this reciprocal communication is due the ready inflation of the organ, not only from the veins, but also from an aperture on any part of its surface. 2. The venous orifices, less numerous than the communicating, and scattered here and there, a single vesicle sometimes containing two or three, at others one cannot be found in a group of several vesicles. They are of a circular or elliptical shape, one-twelfth of a millimetre in diameter, and are the absorbing mouths of veins. Between the vesicles are septa, or intervesicular spaces, formed by the separation of their lining membrane, and containing the splenic glands and vessels, their size varying with the greater or less repletion of the organ, but generally bearing the relation of 2 to 3 to the vesicular part. The investing membrane, forming the walls of the cells, is continuous through the whole extent of the spleen, and may be considered as one homogeneous whole, divided into numerous little ampullæ. It is supported by vessels and glands, and forms a sort of frame-work to the organ. This membrane is very complicated in its structure, and cannot therefore be considered, as by Malpighi, to be a simple dilatation of the internal tunic of the veins.

The splenic arteries and veins, running side by side, are directed towards the periphery of the spleen, the veins being pierced in their whole extent by little circular apertures, leading into the smaller intervesicular branches, which are distributed to the splenic glands and the membranes of the vesicles. The small vessels project into the cavities of the vesicles in a peculiar manner to reach the floating corpuscules, and present the appearance of a bunch of grapes. Lastly, all the small vessels of the spleen, in the turgid state, present numerous dilatations and contractions, so as to give them a marked knotted appearance.

The corpuscules are small bodies floating in the cavities of the vesicles, to the walls of which they are attached in a pediculated manner, by the extreme branches of the blood-vessels and lymphatics. They are about fourteen or fifteen times the size of the blood-globules. By the granulo-capillary basis is intended to be represented the two structures placed beneath the vesicular membrane, viz. spherical pale granules, or glands, four or five times the diameter of the blood-globules, and the capillary net-work of veins, arteries, and lymphatics.

The splenic liquid, which appears to be elaborated by the floating corpuscules and the granulo-capillary basis, is deposited in the cavities of the vesicles, or taken up by the absorbing veins or their walls. It is thick, viscous, of a reddish-brown color, and under the microscope appears to consist of globules suspended in a yellowish unctuous fluid. 1. Lenticular globules, some of which are surrounded with a red border, and appear to differ very little from ordinary blood-globules; others being colorless. 2. Whitish globules, irregular in form and size, and resembling those found in the chyle and lymph.

The splenic glands, so far as actual volume and consistence are concerned, form the principal organic element of the spleen. They fill, with the ramifications of the vessels, the intervesicular spaces. Their diameter is a quarter of a milli metre. They are united by bands of a similar nature with themselves, which extend over every part of the spleen. From the afferent and efferent lymphatic trunks, and the dispositions of the vessels about them, M. Bourgery is convinced that they are simply minute lymphatic glands. The lymphatic vessels are extremely numerous in the spleen, and in many situations remarkable in their structure, being enlarged at intervals, and, in addition to their valves, divided in their interior by septa, which gives them a sort of rudimentary glandular structure, and shows them to be not merely canals for transport, but also, in some degree, organs of elaboration.

M. Bourgery considers the spleen as an apparatus for the elaboration of the blood, and draws an analogy between it and the lymphatic glands; observing that if, in relation to its anatomical structure, we may define the spleen as a vast lymphatico-sanguineous gland, on the other hand, lymphatic glands generally may be considered, to a certain extent, as small spleens appended to different parts of the circulatory apparatus. In treating of the intimate structure of these glands, we shall see how far the idea of the conformity of these two sorts of organs, evident so far as the glandular structure of the spleen is concerned, is borne out by the internal organization of the canals of the lymphatic glands.—*Ibid, from Gazette Médicale.*

